# Microglial‐targeted nSMase2 inhibitor fails to reduce tau spread in the animal model of Alzheimer’s disease

**DOI:** 10.1002/alz.091930

**Published:** 2025-01-09

**Authors:** Meixiang Huang, Carolyn Tallon, Xiaolei Zhu, Kaitlyn Huizar, Silvia Piccirillo, Ajit G Thomas, Lukas Tenora, Wathsala Liyanage, Francesca Roda, Alice Gualerzi, Rangaramanujam M Kannan, Marzia Bedoni, Rana Rais, Barbara S Slusher

**Affiliations:** ^1^ Johns Hopkins University School of Medicine, Baltimore, MD USA; ^2^ Università Politecnica delle Marche, Ancona Italy; ^3^ Johns Hopkins University, Baltimore, MD USA; ^4^ IRCCS Fondazione Don Carlo Gnocchi ONLUS, Milan Italy; ^5^ Laboratory of Nanomedicine and Clinical Biophotonics (LABION), Milan Italy

## Abstract

**Background:**

Cognitive decline associated with Alzheimer’s disease (AD) correlates with hyperphosphorylated tau (pTau) propagating between neurons along networks connected by synapses. It has been hypothesized this transcellular transmission occurs partially by extracellular vesicles (EVs). Both genetic and pharmacological inhibition of nSMase2 has been found to inhibit EV biogenesis and pTau propagation. However, the lack of suitable nSMase2 inhibitors for clinical development has been a challenge. In our lab, a highly selective and nM potency nSMase2 inhibitor, termed DPTIP, was identified through a high‐throughput campaign. However, it has poor oral pharmacokinetics (PK), modest brain penetration, and rapid clearance, limiting its clinical translation. To improve its PK properties, we conjugated it to a hydroxyl‐PAMAM dendrimer delivery system, creating dendrimer‐DPTIP (D‐DPTIP), which selectively targets microglia. In previous studies, using a murine AAV‐hTau brain injection propagation model, we showed that administration of D‐DPTIP significantly inhibited tau spread. In this study we extended those studies to evaluate D‐DPTIP in Tau P301S (PS19) transgenic mice.

**Method:**

PS19 mice were chronically dosed for 20 weeks. Subsequently cognitive function was evaluated using the Y‐maze and Novel Object Recognition tests, and hippocampal volume was measured through MRI. Total tau and pTau levels were quantified by immunoblotting. Immunofluorescent staining and fluorescence‐activated cell sorting (FACS) were employed to identify cell types in the brain responsible for D‐DPTIP internalization. nSMase2 target engagement assays were conducted in both CD11b+ (microglial) and CD11b‐ (non‐microglial cells). Surface plasmon resonance imaging (SPRi) was used to characterize the origin of brain extracellular vesicles (EVs) released into plasma in PS19 mice ± D‐DPTIP treatment.

**Result:**

Total tau, pTau, cognitive deficits, and hippocampal volume loss were not altered by D‐DPTIP treatment in the PS19 mice. D‐DPTIP was found to be co‐localized with microglia and selectively decreased nSMase2 activity in the CD11b+ cells. SPRi analysis showed a decrease in levels of activated microglia‐derived plasma EVs following treatment.

**Conclusion:**

D‐DPTIP selectively targets and inhibits nSMase2 activity in microglia. Consequently, in the AAV‐hTau seeded model where microglial EVs play a central role, D‐DPTIP successfully inhibits tau propagation. However, its effectiveness is limited in PS19 mice, where neuronal‐mediated tau propagation is thought to be predominant.

